# Journeys towards accessing an autism diagnosis and associated support: A survey of families of autistic children in Ecuador

**DOI:** 10.1177/13623613241281029

**Published:** 2024-09-28

**Authors:** Paulina Buffle, Thalia Cavadini, María de Lourdes Ortega, Cristina Armijos, Patricia Soto, Edouard Gentaz, Laura Crane

**Affiliations:** 1 University of Geneva, Switzerland; 2 Asociación de Padres y Amigos para el Apoyo y la Defensa de los Derechos de las Personas con Autismo del Ecuador (APADA); 3 Universidad de los Hemisferios, Ecuador; 4 Red Autismo Ecuador; 5 University of Birmingham, UK

**Keywords:** autism, diagnosis, Ecuador, families, parents, support

## Abstract

There has been limited research on families of autistic children in Ecuador, especially in relation to their experiences of accessing a diagnosis and associated support. In this study, we surveyed 767 families about their diagnostic and post-diagnostic journeys. Of these, 651 families had children who had received a formal autism diagnosis. In this group, we found that families usually suspected that their children may be developing atypically when they were between 6 and 48 months of age; families typically consulted a professional shortly after noticing signs of atypical development (e.g. within 6 months); most children received a diagnosis before the age of 48 months; and families typically reported consulting four or more different professionals to attain a diagnosis for their children. Families’ feelings and thoughts related to the diagnostic and post-diagnostic journey were often negative and were underpinned by concerns around a lack of information and services. We conclude with recommendations for optimal diagnostic and post-diagnostic support for autistic children and their families in Ecuador.

**Lay abstract:**

There has been much research about the experiences of families of autistic children as they navigate the process of accessing a diagnosis and associated support. However, most of this work has been conducted in Europe, the United States, and Australia. In this study, we examined the experiences of 767 families in Ecuador via an in-depth survey. Of the families we surveyed, 651 had children whose journeys resulted in them receiving a formal autism diagnosis. Most families realized that their children might have developmental differences when they were between the ages of 6 and 48 months, after which they tended to seek support from a professional fairly quickly (i.e. within 6 months). Most families consulted with several different professionals before they accessed a diagnosis for their children, with children tending to receive a diagnosis before the age of 48 months. Families often reported negative emotions around their children’s diagnostic and post-diagnostic journeys, which were commonly related to the lack of information and services available to them. We hope that through gaining a greater understanding of the experiences of families of autistic children in Ecuador, these findings can be used to inform public policies that lead to the development of supports and services that better meet the needs of autistic people and their families in this context.

Early identification, diagnosis and support for autistic children are global priorities (World Health Organization, 2012, 2014), but can be challenging and complex for families to access. A scoping review based on 122 articles, mostly from high-income countries (82%), found that the time between parents’ first concerns about their children’s development and the provision of subsequent diagnoses ranged from 12 to 55 months (Makino et al., 2021). This review also highlighted that most current evidence on families’ diagnostic journeys stems from English-speaking samples and high-income countries. Likewise, while several best practice guidelines have been published to promote timely identification, diagnosis and support for autistic children and their families, these guidelines are mainly from high-income countries, for example, Great Britain (National Institute for Health and Clinical Excellence [NICE], 2011), France (Haute Autorité de Santé, 2018), the United States (Hyman et al., 2020; Volkmar et al., 2014), Australia (Whitehouse et al., 2018) and Canada (Anagnostou et al., 2014; Brian et al., 2019; Zwaigenbaum & Penner, 2018); with some examples from low- to middle-income countries such as Ecuador (Buffle & Naranjo, 2021) and India (Dalwai et al., 2017).

Extrapolating evidence and recommendations on accessing a diagnosis and post-diagnostic support for autistic children and their families from high-income countries to low- and middle-income countries is problematic (Cheng et al., 2023), since services and systems may be very different across these contexts. As one example, a lack of diagnostic services, alongside a lack of reporting of autism diagnoses in official records, may render a potentially sizable proportion of autistic individuals ‘invisible’. Second, family characteristics are known to affect access to diagnoses and subsequent services differently across contexts. For example, living in semi-urban or rural settings has been associated with later diagnosis in high, low- and middle-income countries (Antezana et al., 2017; Bent et al., 2015). However, family characteristics may have a different impact in other cultural contexts and health systems.

A survey conducted across six Latin American countries (Brazil, Argentina, Chile, Uruguay, Venezuela, and the Dominican Republic), by the Latin American Autism Spectrum Network (*Red Espectro Autista Latinoamerica*) revealed that despite parents expressing their initial concerns about their children’s development around 22 months of age, the actual diagnosis occurred 24 months later (Montiel-Nava et al., 2020). As such, the mean age of diagnosis typically coincided with the beginning of formal schooling. Among families in these countries whose children had received a formal diagnosis of autism, their primary concerns were (1) the need for increased awareness of autism among the community and (2) the need for improvements in the education system (Paula et al., 2020).

Ecuador is an Upper Middle-Income Country characterized by income inequality across regions and ethnic groups. The prevalence of autism and the ‘age of diagnosis’ have not been definitively assessed, nor are children routinely screened for autism (Buffle et al., 2022). A preliminary study in the capital city, Quito, found that 0.11% of 453 students in mainstream schools (aged 5–15 years) had an autism diagnosis (Dekkers et al., 2015). More recently, Ecuador’s Ministry of Public Health (2017) suggested that the prevalence of autism was 0.28% (0.18%–0.41%) in children aged 5 years or less, akin to 1266 children. To our knowledge, the reasons why those estimates are considerably lower than international prevalence figures (e.g. Zeidan et al., 2022) have not been formally studied. However, difficulties in accessing services likely result in some children not receiving a formal diagnosis of autism from a health professional. For example, in some cases, health or educational professionals may suggest that a child ‘might be autistic’ despite them not providing an assessment or formal diagnosis. In other cases, parents may engage with online resources and have discussions with those around them (e.g. family members, professionals), leading them to understand that their child may be autistic, in the absence of a formal diagnosis.

Ecuador’s healthcare system comprises both public and private sectors. The public sector includes the Ministry of Public Health (MSP), the Ministry of Economic and Social Inclusion (MIES), and municipal health services, and various social security institutions, including the National Social Security Institute (IEESS). The MSP delivers healthcare services to the entire population, while the MIES and municipalities run health programmes and facilities that cater to uninsured individuals. In contrast, the private sector includes for-profit organizations such as hospitals, clinics, dispensaries, offices, pharmacies and prepaid medical companies (Lucio et al., 2011). In 2020, public expenditure on health accounted for just 4.97% of the gross domestic product (GDP) and 13.91% of total public expenditure (World Health Organization, 2016).The National Health System in Ecuador recognizes several types of disabilities, including auditory, physical, intellectual, language, psychosocial and visual (Ministry of Public Health, 2020). While autism is not explicitly mentioned under these categories, it is included in a separate list of ‘catastrophic, rare, and orphan diseases’ that ensures access to certain benefits for cases deemed ‘serious’ (Ministry of Public Health, 2012).

Ecuador is part of the International Convention on the Rights of Persons with Disabilities (UN General Assembly, 2007). It has integrated these rights into national regulation, article 33 of the Law on Disabilities (2012) (National Assembly of the Republic of Ecuador, 2012), which warrants, for example, free access to medicines, equipment, educational adaptations and support. While a 10-year health plan (2022–2031) has been developed in Ecuador (Pan American Health Organization, 2021), a plan for autism or other neurodevelopmental conditions does not exist (individually or within the broader health plan).

Families typically access support for autistic children by obtaining a ‘disability card’ and, more recently, ‘disability’ status is registered in children’s identity cards (Ministry of Public Health, 2020). The disability level is rated as a percentage, with a minimum of 30% required to qualify for support. However, the method for calculating the disability percentage is poorly specified. According to this regulation, individuals can only apply for disability status through evaluations, qualifications and requalifications conducted within the public health network by professionals trained and accredited by the Ministry of Public Health. Consequently, families whose children do not have a diagnosis in the public system are unable to access publicly funded services. The lack of services for individuals without an official diagnosis may lead these families to seek support and information from charitable or for-profit associations.

To date, limited research has examined the identification, diagnosis and support of autistic children in Ecuador. In one of the few studies on this topic, Buffle et al. (2022) conducted a survey with 153 paediatric professionals (e.g. paediatricians, family doctors) about their experiences of, and self-efficacy in, screening children for autism. Encouragingly, more than 90% of participants were aware of the need for an autism-specific screening tool, and more than 75% knew where to access one. However, participants also identified a range of factors that they perceived to negatively influence routine identification practices, including lack of time, a lack of resources to refer to, and concerns about unnecessarily alarming families by screening for autism. Furthermore, three-quarters of the sample reported a lack of training on the identification of autism, and a similar proportion wanted more knowledge to be able to confidently identify autistic children. Notably, almost all participants (98%) wanted more training around autism.

Despite Buffle et al.’s study providing some clues around the challenges faced by families of children in Ecuador when seeking an autism diagnosis and associated support, little is known from the perspectives of families themselves. In this study, we sought to conduct a comprehensive examination of families’ journeys towards accessing a diagnosis and post-diagnostic support for autistic children in Ecuador. Our specific aims were (1) to examine families’ journeys towards their children’s autism diagnosis, (2) to examine respondents’ report of the support available for families and their children, and (3) to gather families’ thoughts and feelings about the process of accessing a diagnosis and associated support.

## Method

### Community involvement statement

Our study was designed and developed in collaboration with representatives from the Ecuadorian Parents’ Association for the Defence of the Rights of Persons with Autism (APADA). APADA representatives helped shape the research study, including advising on the research questions, methods, and implications of the work, and they also facilitated recruitment. M.d.L.O. from APADA is credited as a co-author of the paper. A panel of Ecuadorian community members (including parents and professionals) also provided input on the survey design, but there were no autistic contributors to the project.

### Participants

The research was open to adult family members of at least one child that they considered to be autistic. We did not confine participation to those whose children had a formal diagnosis of autism for two reasons. First, our community partners, APADA, highlighted the difficulties families often faced in accessing an autism diagnosis, and we wanted to capture this landscape accurately within our research. Second, we were interested in learning about journeys to accessing an autism diagnosis, which would not necessarily have resulted in children receiving a formal diagnosis. However, all participating families reported that their children were autistic (whether formally diagnosed or not). All families needed to reside in Ecuador at the time of the survey to be eligible to participate.

### Materials

Administered via the online survey platform Qualtrics, our bespoke questionnaire was adapted from previous surveys of families’ experiences of accessing a diagnosis and post-diagnostic support for their autistic children (Crane et al., 2016; Howlin & Asgharian, 1999) and from reviewing literature on factors associated with an autism diagnosis (Bent et al., 2015; Coo et al., 2012; Daniels & Mandell, 2014; Fountain et al., 2011; Jacobs et al., 2020; Paula et al., 2020). A panel of Ecuadorian parents and professionals provided suggestions for adapting the questionnaire to make it maximally informative. The final questionnaire (see Supplementary Materials 3) was divided into five sections, collecting information on (1) the respondent, (2) their autistic child, (3) initial concerns and first consultations, (4) the diagnostic process, and (5) post-diagnosis support. While not relevant to the research questions (and therefore not presented here), the survey ended with a question on the child’s interests, abilities and strengths, to end the survey on a positive note.

### Procedure

Ethical approval was obtained from the Research Ethics Committee at the University at which the first author is affiliated. APADA facilitated recruitment. At the time of the study, APADA had 60 formal members along with 1250 beneficiaries (parents and autistic individuals who joined their social media groups, conferences and meetings). Among these members and beneficiaries, some families have children with a formal autism diagnosis, while others think that their child might be autistic. APADA provided information about the current research to all its members and beneficiaries. A statement was also issued on regional and national radio stations (Radio Municipal, Pichincha, Radio Católica Nacional) requesting that interested parents of autistic children contact APADA to obtain information about the research. APADA additionally invited six other parents’ associations from different provinces (Guayas, Manabí Azuay, Milagro, Loja, and Chimborazo) and professional associations (Asociación de Fonoaudiólogos, Asociación de Psicólogos Infantiles, Asociación de Terapistas Ocupacionales) to share the research. Information about the research was also published on the web page of the National Council on Disability. The telephone number of two representatives of APADA was provided for parents who had difficulties completing the questionnaire (e.g. due to lack of Internet access).

All eligible respondents provided informed consent before completing the survey anonymously between 19 May and 16 June 2022. Data are securely stored on an institutional computer protected by a password.

### Data analysis

Data collected through closed-ended questions were largely presented descriptively (i.e. numbers/percentages of responses), both for the total sample and for those with and without a formal autism diagnosis. Pearson Chi-square tests were conducted to determine if there were differences in the distribution of responses between those with and without a formal diagnosis. Furthermore, multiple linear regressions were performed with the following independent variables: estimated family income (coded from 1 = very low, to 6 = high) and living location (rural or urban). One regression was used with age of first concerns as the dependent variable and another was performed with the age of formal diagnosis as the dependent variable.

Data collected through open-ended questions were analysed using conceptual content analysis to identify categories corresponding to ideas or expressions that were common among participants (Elo & Kyngäs, 2008; Kvale, 2008). Before coding, all responses were read several times by Spanish speaker authors (P.B., M.d.L.O., P.S., C.A.) to identify regional colloquial expressions or locutions, which were clarified by Ecuadorian co-authors. Then, the first and third authors jointly proceed to the initial coding stage by assigning labels to words or phrases representing essential and recurring themes in each response, working together to iteratively develop categories. The unit of coding was single words when participants provided a sole word or phrase. Once the final categories were agreed upon between the two authors, the data was independently coded into the new categories by the two authors, and respective coding were compared for all codes. A good level of agreement between the coding was found (85%, 97% and 90% for the first, second and third open questions, respectively). Disagreements were resolved via discussion. For reporting, the first author translated quotes from Spanish to English. Another author (CA) (fluent in Ecuadorian Spanish and English) reviewed the translations to ensure the accuracy of expression. All three authors involved had received training in and had experience conducting content analysis (e.g. during research and/or professional training). The quality and rigour of the analysis were enhanced by following the recommendations of Elo et al. (2014).

## Results

### Survey respondents

A total of 1043 respondents engaged with the survey, although 276 were excluded: 3 did not provide consent, 177 were incomplete (i.e. completed less than 90% of the first six sections of the survey) and 96 were identified as spam. The final sample comprised 767 respondents (87% female, 13% male; 74% of whom were between 30 and 49 years of age). As per Table 1, respondents were predominantly parents of an autistic child (81% mothers, 11% fathers) who did not have other children with Special Education Needs and Disabilities (SEND) under their care (85%). A quarter were single parents, and most were members of support groups (65%). A minority of respondents identified as autistic themselves (10% had an autism diagnosis, and 22% thought they might be autistic). Employment statuses varied (with 57% in some form of employment while 41% were not working), as did family income (although most identified as middle/low earners).

**Table 1.** Participants’ characteristics.

		Total sample (*n* = 767)	With a formal autism diagnosis (*n* = 651)	Without a formal autism diagnosis (*n* = 116)	Pearson χ^2^, *p*-value
		*n* (%)	*n* (%)	*n* (%)	
Relationship to the autistic child					
	Mother	624 (81%)	532 (82%)	92 (80%)	χ^2^(4) = 6 *p* = 0.199
	Father	82 (11%)	71 (11%)	11 (9%)	
	Other family member (e.g. aunt/uncle, grandparent, sibling)	61 (8%)	48 (7%)	13 (11%)	
Age					
	18–29 years old	99 (13%)	81 (12%)	18 (16%)	χ^2^(25) = 30 *p* = 0.224
	30–39 years old	313 (41%)	266 (41%)	47 (41%)	
	40–49 years old	251 (33%)	221 (34%)	30 (26%)	
	50–59 years old	77 (10%)	62 (10%)	15 (13%)	
	⩾ 60 years old	26 (3%)	20 (3%)	6 (5%)	
	No answer (NA)	1 (<1%)	1 (<1%)	0	
Gender					
	Female	669 (87%)	567 (87%)	102 (88%)	χ^2^(4) = 6 *p* = 0.199
	Male	98 (13%)	84 (13%)	14 (12%)	
Sole caregiver (e.g. single parent)					
	Yes	200 (26%)	172 (26%)	28 (24%)	χ^2^(4) = 6 *p* = 0.199
	No	565 (74%)	479 (74%)	86 (74%)	
	NA	2 (<1%)	0	2 (2%)	
Ethnic origin					
	Mestizo	696 (91%)	592 (91%)	104 (90%)	χ^2^(12) = 15 *p* = 0.241
	White	41 (5%)	35 (5%)	6 (5%)	
	Afro-descendant	20 (3%)	17 (3%)	3 (3%)	
	Indigenous	9 (1%)	6 (1%)	3 (3%)	
	NA	1 (<1%)	1 (<1%)	0	
Current employment situation					
	Employed in a full-time work	257 (34%)	228 (35%)	29 (25%)	χ^2^(49) = 56 *p* = 0.229
	Employed in part-time work	59 (8%)	51 (8%)	8 (7%)	
	Freelance work	116 (15%)	93 (14%)	23 (20%)	
	Looking for a job	66 (9%)	56 (9%)	10 (9%)	
	Stopped working to take care of autistic child	137 (18%)	115 (18%)	22 (19%)	
	Not working: needs covered by family	101 (13%)	84 (13%)	17 (15%)	
	Not working because of a disability	5 (1%)	5 (1%)	0	
	NA	26 (3%)	19 (3%)	7 (6%)	
Highest level of education					
	Master or doctoral degree	107 (14%)	99 (15%)	8 (7%)	χ^2^(30) = 35 *p* = 0.243
	University undergraduate degree	392 (51%)	329 (51%)	63 (54%)	
	Technical diploma	50 (7%)	43 (7%)	7 (6%)	
	Craft certificate	11 (1%)	8 (1%)	3 (3%)	
	Secondary school	199 (26%)	164 (25%)	35 (30%)	
	Primary school	6 (1%)	6 (1%)	0	
	NA	2 (<1%)	2 (<1%)	0	
Estimated monthly family income					
	<$425	180 (23%)	149 (23%)	31 (27%)	χ^2^(25) = 30 *p* = 0.224
	$425–$750	219 (29%)	192 (29%)	27 (23%)	
	$750–$2000	186 (24%)	161 (25%)	25 (22%)	
	$2000–$5000	50 (7%)	40 (6%)	10 (9%)	
	>$5000	6 (1%)	6 (1%)	0	
	NA	126 (16%)	103 (16%)	23 (20%)	
Monthly income perceived					
	Very low income	73 (10%)	59 (9%)	14 (12%)	χ^2^(30) = 35 *p* = 0.243
	Low income	170 (22%)	136 (21%)	34 (29%)	
	Middle-low income	218 (28%)	193 (30%)	25 (22%)	
	Middle income	268 (35%)	232 (36%)	36 (31%)	
	Middle-high income	35 (5%)	28 (4%)	7 (6%)	
	High income	3 (<1%)	3 (<1%)	0	
	Very high income	0	0	0	
Participant has an autism spectrum diagnosis					
	Yes	78 (10%)	65 (10%)	13 (11%)	χ^2^(4) = 6 *p* = 0.199
	No	686 (89%)	586 (90%)	100 (86%)	
	NA	3 (<1%)	0	3 (3%)	
Participant ‘thinks’ they might be autistic					
	Yes	170 (22%)	146 (22%)	24 (21%)	χ^2^(4) = 6 *p* = 0.199
	No	594 (77%)	504 (77%)	90 (78%)	
	NA	3 (<1%)	1 (<1%)	2 (2%)	
Other children or people with special needs under their care					
	Yes	117 (15%)	96 (15%)	21 (18%)	χ^2^(4) = 6 *p* = 0.199
	No	650 (85%)	555 (85%)	95 (82%)	
Member of a support group					
	Yes	501 (65%)	429 (66%)	72 (62%)	χ^2^(4) = 6 *p* = 0.199
	No	264 (34%)	221 (34%)	43 (37%)	
	NA	2 (<1%)	1 (<1%)	1 (1%)	

The 767 respondents were subdivided into two groups according to whether their child had been formally diagnosed as autistic (*n* = 651) or not (*n* = 116). Socio-demographic information was similar across the two groups (see Supplementary Material 1 for further details)

Characteristics of the children with (*n* = 651) and without (*n* = 116) a formal autism diagnosis are presented in Table 2. In summary, most children were male (81%), under the age of 10 years (65%), from urban areas (93%) and had one or two siblings (64%). These characteristics were similarly distributed across the subgroups.

**Table 2.** Children’s characteristics.

		Total sample (*n* = 767)	With a formal autism diagnosis (*n* = 651)	Without a formal autism diagnosis (*n* = 116)	Pearson χ^2^, *p*-value
		*n* (%)	*n* (%)	*n* (%)	
Age					
	<2 years old	11 (1%)	10 (2%)	1 (1%)	χ^2^(72) = 80 *p* = 0.242
	2–3 years old	147 (19%)	111 (17%)	36 (31%)	
	4–6 years old	224 (29%)	198 (30%)	26 (22%)	
	7–9 years old	115 (15%)	104 (16%)	11 (9%)	
	10–12 years old	92 (12%)	83 (13%)	9 (8%)	
	13–15 years old	57 (7%)	49 (8%)	8 (7%)	
	16–18 years old	37 (5%)	35 (5%)	2 (2%)	
	19–29 years old	71 (9%)	54 (8%)	17 (15%)	
	⩾ 30 years old	7 (1%)	4 (<1%)	3 (3%)	
	NA	6 (1%)	3 (<1%)	3 (3%)	
Residence					
	Urban	716 (93%)	610 (94%)	106 (91%)	χ^2^(4) = 6 *p* = 0.199
	Rural	47 (6%)	40 (6%)	7 (6%)	
	NA	4 (<1%)	1 (<1%)	3 (3%)	
Gender					
	Male	624 (81%)	532 (82%)	92 (79%)	χ^2^(6) = 8 *p* = 0.238
	Female	139 (18%)	117 (18%)	22 (19%)	
	Other	1 (<1%)	1 (<1%)	0	
	NA	3 (<1%)	1 (<1%)	2 (2%)	
Siblings					
	0 sibling	228 (30%)	187 (29%)	41 (35%)	χ^2^(16) = 20 *p* = 0.446
	1 sibling	353 (46%)	304 (47%)	49 (42%)	
	2 siblings	138 (18%)	122 (19%)	16 (14%)	
	3 or more siblings	40 (5%)	32 (5%)	8 (7%)	
	NA	8 (1%)	6 (1%)	2 (2%)	

There were no significant differences between the characteristics of the groups with and without a formal autism diagnosis (see Tables 1 and 3), indicating that these groups were broadly similar on key demographic variables.

**Table 3.** Type of professional consulted, diagnostic decision after the first consultation, co-occurring condition diagnosed and difficulties not formally diagnosed.

	*n* (%)
Professional who provided the formal diagnosis of autism (*n* = 651)	
Neurologist (child, adult)	280 (43%)
Psychologist (psychologist, child psychologist, child neuropsychologist, neuropsychologist)	267 (41%)
Psychiatrist (child, adult)	57 (8%)
Paediatrician	11 (2%)
Community health doctor	2 (<1%)
Not sure	3 (<1%)
Other	31 (5%)
Professional’ diagnostic decision after the first consultation (*n* = 651)	
Autism	332 (51%)
Global developmental delay	46 (7%)
Pervasive developmental disorder	37 (6%)
Language disorder	34 (5%)
Other open responses	
There was not a clear diagnosis	33 (5%)
Some non-specified difficulty	20 (3%)
ADHD	13 (2%)
Epilepsy	4 (1%)
NA	132 (20%)
Co-occurring conditions diagnosed (*n* = 246; 302 responses recorded)	
A physical disability (e.g. epilepsy, hearing and visual impairments) and motor delay	103 (34%)
A behavioural condition (ADHD, conduct disorder, Tourette syndrome)	80 (26%)
A learning disorder (incl. dyslexia and general learning disabilities) or an intellectual delay	68 (23%)
An affective condition (incl. depression and anxiety)	27 (9%)
A genetic condition (e.g. fragile-x syndrome)	10 (3%)
A mental health condition (e.g. bipolar disorder, OCD, schizophrenia)	9 (3%)
A language disorder (no language or speech difficulties)	5 (2%)
Co-occurring conditions or difficulties not formally diagnosed (*n* = 678 parents; 1042 responses recorded)	
Behaviour difficulties	332 (22%)
Atypical sensory processing	251 (17%)
Language delay or absence	225 (15%)
Sleep difficulties	194 (13%)
Eating disorders	150 (10%)
Digestive difficulties	148 (10%)
Intellectual difficulties	140 (9%)
Other, including affective, motor and learning difficulties	37 (3%)

### Initial concerns and first consultations

#### Early signs

In our sample (*n* = 767), most families noticed signs suggesting that their children were developing atypically before the age of 4 years (*n* = 697; 91%) and most frequently when the child was between 12 and 24 months (*n* = 361; 47%). The top three signs were in the domains of language (*n* = 507; 66%), lack of responding to their name (*n* = 426; 56%), and social interaction (421; 55%) (see Supplementary Materials 4 for full details). Families searched for professional help immediately (*n* = 205; 31%) or within six months (*n* = 490; 75%), mainly heading to professionals in private practices (*n* = 467; 61%) as opposed to national health services or services covered by the public insurance system (*n* = 236; 31%). Parents who did not seek a consultation (*n* = 18; 16%) reported not doing so because they did not know where to go. Note that these patterns were common among families of autistic children who had and had not received a formal diagnosis, and there did not appear to be any demographic differences across these groups. Next, we present data only from the 651 respondents who reported that their child received a formal diagnosis of autism to map their journeys to diagnosis.

#### First professional consulted

The 651 families whose children received a formal diagnosis of autism most commonly had a first consultation with a paediatrician (*n* = 219; 34%), neurologist (*n* = 171; 26%), or neuropsychologist (*n* = 4; 23%). The remaining participants approached other professionals (e.g. family doctor or community health doctor). In many cases (*n* = 294; 45%), the professional that they approached provided their children with a diagnosis (often of autism). For 137 families (21%), the professional that they consulted with made a referral to another professional (e.g. neurologist, psychiatrist), and for 194 families (30%), the professional that they consulted with either had a ‘wait and see’ attitude or did not take any action (see Table 3).

### The diagnostic process

#### Age at diagnosis

A total of 205 children (31%) received a diagnosis before the age of 36 months, while 249 children (38%) were between the ages of 3–4 years, 81 children (12%) were between the ages of 5–6 years and all other children were 7 years of age or older (see Supplementary Materials 5 for full details).

#### Professionals providing the diagnosis

The formal diagnosis was mostly provided by psychologists (*n* = 267, 41%) or neuropaediatricians (*n* = 226, 35%). Other diagnoses stemmed from other professionals including neurologists, psychiatrists and community doctors (see Table 3).

#### Number of professionals consulted to reach a formal diagnosis

Many families reported that they consulted a second or further professional to obtain a diagnosis: 110 families (20%) consulted two professionals, 174 (32%) consulted three, 242 (45%) consulted more than four professionals and 14 (3%) did not answer. Of these families, 294 (45%) reported that they had received a diagnosis at the first consultation, indicating that some of the parents who consulted another professional had already received a diagnosis of autism for their children.

#### Co-occurring conditions diagnosed and additional needs identified

Of the 636 respondents to this question, 390 (61%) reported that their child did not have a diagnosis of a co-occurring condition. The remaining 246 (39%) provided 302 responses, which are provided in Table 3. In summary, the most common diagnoses were a behavioural condition, physical disability, and a learning disorder or intellectual delay.

Families were also asked about any co-occurring conditions or difficulties that they felt that their children had, but which had not been formally diagnosed. As seen in Table 3, 678 participants provided data to this question, and the most commonly reported conditions or difficulties were behaviour difficulties, atypical sensory processing, and issues with language.

#### Funding and geographic location

Diagnoses were mainly paid for by the families themselves (*n* = 439; 67%), but families also reported the diagnoses being paid for by the national health network (*n* = 102; 16%) or the public insurance system (IESS) (*n* = 46; 7%). A minority of families (*n* = 64; 10%) relied on alternative options such as charities, institutional/private insurance systems, or private professionals who did not charge for their services. Most participants received the diagnosis for the children in the capital province, Pichincha (60%), or in Guayas (20%). The remaining families received their diagnosis in one of the other 14 provinces (see Supplementary Materials 6).

#### Predicting first concerns and age of diagnosis

Two multiple linear regressions were conducted after controlling for assumptions of normality, homoskedasticity, and linearity to test whether estimated family income and living location predicted (a) the age of first concerns and (b) the age at which the formal diagnosis was made. Regressions were not statistically significant (see Table 4a and 4b).

**Table 4.** Two multiple linear regression (MLR) with socioeconomic status and residency as predictors of (a) initial parental concerns and (b) the age of formal diagnosis.

(a) MLR with estimated family income and living location as predictors of initial parental concerns				
	Age at first concerns			
	Standardized beta (*β*)	*t*(759)	*p*-value	*R* ^2^
				0.005
Estimated family income	0.070	1.938	0.053	
Living location	0.001	0.035	0.972	
(b) MLR with estimated family income and living location as predictors of the age of formal diagnosis				
	Age at formal DX			
	Standardized beta (*β*)	*t*(646)	*p*-value	*R* ^2^
				0.0002
Estimated family income	0.015	0.378	0.706	
Living location	−0.003	−0.088	0.930	

#### Emotions and thoughts when receiving a diagnosis and after diagnosis

**Figure 1.**
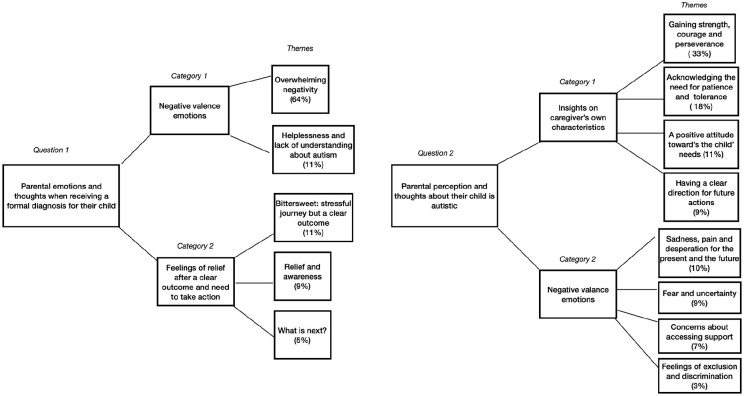
Thematic map.

Analysis of open-ended responses concerning participants’ thoughts and feelings when receiving a diagnosis (*n* = 630) were organized into two categories and five themes (see Table 5a). Analysis of open-ended responses concerning perceptions and understanding of their children’s autism (*n* = 621; 1031 responses recorded) were organized into two categories and eight themes (see Table 5b). See also Figure 1.

**Table 5.** Qualitative analysis of parental thoughts and feelings when receiving their child’s diagnosis and their perceptions of their child’s autism.

a. Question: Please choose a few words to tell us what your emotions and thoughts were when you received the diagnosis (*n* = 630)			
Categories and themes	*n*; %		Examples
**Category 1: Negative valence emotions**			
** Theme 1: Overwhelming negativity.** This theme include vocabulary related to shock, grief, and personal impact, mostly related to the challenge of addressing the child’s immediate needs, alongside the anxiety about their child future well-being, especially in a time when parents are no longer present to provide care.	**(***n* **=** **403; 64%)**		“It was something you could see coming, but you don’t want to accept because it is so difficult.” “I felt sadness and devastated not knowing what he will become if I am not longer here”
** Theme 2: Helplessness and lack of understanding about autism.** Parent’s words express limited knowledge about a complex and diverse condition, combined with the perceived lack of immediate solutions or support.	**(***n* **=** **69; 11%)**		“I felt despair, because I didn’t know anything about ASD.” “I felt powerless knowing that the country doesn’t have professionals to provide guide, or to explain to me what to do to help my child.”
**Category 2: Feelings of relief after a clear outcome and need to take action**			
** Theme 3: Bittersweet: a stressful journey but a clear outcome.** This theme captures the intense and emotional rollercoaster faced by caregivers, marked by uncertainty, emotional strain, lack of information, and the need to make significant decisions. Despite the stress, the diagnosis ultimately provides a resolution, leading to a clear and actionable outcome that empowers caregivers to take further actions to support their child.	**(***n* **=** **69; 11%)**		“It was very stressful, but I felt relieved to receive the diagnosis.” “My world came crashing down; we didn’t know which path to take, we didn’t know much about the subject, but we were ready to do anything to help our son.”
** Theme 4: Relief and awareness**. A period of uncertainty and confusion comes to an end, bringing a sense of relief and a more positive outlook This relief stems from clarity provided by finally understanding what is affecting their child, alleviating the stress and anxiety of the unknown. With a definitive answer in hand, caregivers can now engage in focus planning and action.	**(***n* **=** **57; 9%)**		I felt relief because with a diagnosis I could learn what I should do to help him and understand him.” “Peace of mind because now we can learn about his condition and now, we can help him and better understand him.”
a. Question: Please choose a few words to tell us what your emotions and thoughts were when you received the diagnosis (*n* = 630)			
Categories and themes	*n*; %		Examples
** Theme 5: What is next?** Parents clearly express a need for resources and guidance to learn how to support their child as effectively and quickly as possible Many participants use verbs related to empowerment, emphasizing their desire to take an active role in their child’s care. Additionally, some parents highlight the importance of engaging with qualified professionals, often voicing frustration over the lack of knowledge about autism among many in the field.	**(***n* **=** **32; 5%)**		“I felt the need to ask what we could do, and immediately started to study about autism and to learn how to help him.” “Well, I thought that now I know what my daughter has and that I must seek to learn in order to help and understand her because professionals here don’t know what to do, they lack knowledge.”
b. Question: Please choose a few words to explain what it means to you that your child has autism (*n* = 621; 1031 responses recorded) – Examples from different participants			
**Category 1: Insights on caregiver’s own characteristics**			
** Theme 1.1: Gaining strength, courage, and perseverance to fight for their child.** This theme highlights the process of becoming more resilient and committed in order to ensure the child’s well-being and success, despite any obstacles that may arise.	**(***n* **=** **341; 33%)**	“It (autism) represented being prepared to fight every day to be able to educate him and move him forward.” “For me having a child with autism represents an everyday effort to fight, never give up, be more empathetic”	
** Theme 1.2: Acknowledging the need for patience and tolerance.** Recognizing that situations might require an understanding and an accepting approach.	**(***n* **=** **185; 18%)**	“It means that I need to take care of her, and I need to do so with much more patience and understanding” “Patience and acceptance and of differences and challenges.”	
** Theme 1.3: Having a positive attitude towards the child’s needs.** This theme refers to an optimistic and supportive outlook on the child’s requirements. It involves appreciating the child’s presence and viewing him as a valuable and enriching part of life.	**(***n* **=** **112; 11%)**	“Now I see it as a great blessing.” “It represents union and love.”	
** Theme 1.4: Having a clear direction for future actions.** This theme involves parents identifying specific steps to advance and address challenges, ultimately leading to positive outcomes or effective solutions to help their child.	**(***n* **=** **93; 9%)**	“It represents the engine to move forward for him. A great responsibility to seek information and support to learn about ASD and thus be able to help my son.” “It means learning about autism to know how to guide him in the best way of respecting and loving him.”	
**Category 2: Emotions with a negative valence.**			
** Theme 2.1: Sadness, pain, and desperation for the present and future.** This theme refers to deep feelings of sorrow, emotional suffering, and distress both in the current moment and when thinking about what lies ahead.	**(***n* **=** **104; 10%)**	“The hardest thing in my life” “It represents pain, frustration, anguish for not knowing what to do”	
** Theme 2.2: Fear and uncertainty.** Refers to feelings of anxiety and apprehension about the future or unknown situations because of not knowing what to expect or what might happen.	**(***n* **=** **90; 9%)**	“A constant concern since he is an adult and has serious behaviour problems and one day I won’t be able to be here for him.” “Anxiety about thinking what will happen when I am no longer here. A great uncertainty for his safety and future.”	
b. Question: Please choose a few words to explain what it means to you that your child has autism (*n* = 621; 1031 responses recorded) – Examples from different participants			
Categories and themes	*n*; %		Examples
** Theme 2.3: Concerns about accessing support.** This theme refers to worries or anxieties about the availability and ability to obtain necessary help or services. This could involve doubts about whether support will be available, adequate, affordable, and provided by trained professionals.	**(***n* **=** **73; 7%)**	e.g. “It is difficult since there are not many schools that accept him or can support him, as well as the cost of therapies” “A full-time hard work and I do not have any support.”	
** Theme 2.4: Feelings of exclusion and discrimination.** Refers to the unfair treatment of individuals by excluding them from activities, opportunities, or rights that others can access, based on the child’s autism.	**(***n* **=** **29; 3%)**	e.g. “It represents having to make of my daughter the most functional and independent person to face a complex and selfish world.” “Society does not understand anything, it only excludes.”	

### Thoughts in relation to post-diagnostic support

Open-ended responses regarding post-diagnostic support were gathered from 413 respondents and were classified into five different categories (see Table 6). These data suggested that key areas to focus on in relation to post-diagnostic support were (1) upskilling professionals in relation to autism (*n* = 201, 47%) (e.g. ‘It is important to mention that there is a lack of training for professionals working with autism’), (2) having more readily available information on autism for families (*n* = 94, 22%) (e.g. ‘Information for parents is scarce, I had to look on my own for therapies’), (3) ensuring there is available intervention/support (*n* = 93, 22%) (e.g. ‘I personally consider that early intervention is fundamental because it helped me to understand my son, they taught me the exercises that I could do with him at home’) and (4) ensuring that meeting the needs of children does not put a strain on family finances (*n* = 40, 9%) (e.g. ‘My financial situation doesn’t allow me to think of anything to help my daughter’).

**Table 6.** Post-diagnostic support for children who received a formal diagnosis and other type of support (non-professional) after diagnosis.

	*n* (%)
Post-diagnostic support for children who received a formal diagnosis (*n* = 651; 998 responses recorded)	
Language therapy	416 (42%)
Early intervention for autism	237 (24%)
Information about their children’s difficulties	205 (21%)
Sensory therapy	194 (19%)
Behavioural intervention for autism	191 (19%)
Motor therapy	167 (17%)
Parental training	103 (10%)
Occupational therapy	35 (4%)
Psychological therapy	16 (2%)
Equine therapy	15 (2%)
Psychorehabilitation/neurorehabilitation	13 (1%)
Psychoeducation	8 (1%)
Hydrotherapy	6 (1%)
Music therapy	6 (1%)
Physio/physical therapy	5 (1%)
Psychiatric care/treatment	4 (<1%)
Medical treatment	2 (<1%)
Other	30 (3%)
Other (non-professional) support after diagnosis (*n* = 575; 1239 responses recorded)	
General tips on internet for raising autistic children	394 (32%)
Being in contact with other families	380 (31%)
Help to obtain a disability card	238 (19%)
Help to gain access to preschool/school institution	103 (8%)
Being in contact with a charity	38 (3%)
State economic aid (bonds)	34 (3%)
Support with the childs’ medical problems	32 (3%)
Family	5 (<1%)
Other	15 (1%)

### Post-diagnostic support

Among 645 participants who responded to questions on post-diagnostic support, the majority (*n* = 466; 72%) reported that they received suggestions for a follow-up for their child from the professional who provided the diagnosis, while 87 (13%) reported that they did not receive any suggestions for follow-up and 92 (14%) were not sure if they did. A total of 580 respondents provided details of who the follow-up appointments were with, and these were reported to be with private practitioners (*n* = 341; 59%), in the network of the national health system (*n* = 131; 23%), or in both (*n* = 10; 19%).

Our sample of 651 participants provided 998 individual responses about the type of support their child received after diagnosis. As participants could provide more than one response to this question, reported percentages exceed 100%. The most common response provided by respondents was language therapy (*n* = 416; 42%), followed by autism-specific early intervention (*n* = 237; 24%) and information being provided about their child’s difficulties (*n* = 205; 21%). However, a variety of options were mentioned. For example, behavioural intervention, motor therapy and parental training were each reported by 10%–19% of the sample, whereas a smaller minority of participants were offered options including equine therapy, hydrotherapy or music therapy (see Table 6).

Details of who suggested the type of post-diagnostic support were provided by 597 respondents, with some families providing more than one response to this question (meaning that reported percentages exceed 100%). These data indicate that suggestions were mainly made by the professional who gave the diagnosis (*n* = 474; 52%), but suggestions were commonly facilitated by the family themselves (*n* = 154; 26%) or by other families of disabled children (*n* = 89; 15%)

Respondents were also asked about the advice and support they received more generally following the diagnosis. A total of 1239 responses were provided from 575 participants. As participants could provide more than one response to this question, reported percentages exceed 100%. These data indicated that most respondents gained their information from the Internet (*n* = 394; 32%) or from other families (*n* = 380; 31%) (see Table 6).

Respondents were asked about the most important sources of support when raising autistic children. Of the 630 respondents, 1064 responses were provided. More than one response to this question (meaning that reported percentages exceed 100%). The responses emphasized the important role of grandparents and extended family (*n* = 576; 54%) but also private professionals (*n* = 147; 13%) and social media (*n* = 107; 10%) (see Supplementary Materials 9 for further details).

## Discussion

Through examining 767 families’ experiences of diagnosis and subsequent support for their autistic children in Ecuador, we highlighted several commonalities with international literature on this topic, but also some important differences. Next, we discuss important aspects of the journey that families of autistic children in Ecuador navigate, before making suggestions to enhance the support currently provided.

It is important to emphasize that this survey was open to families of children who did and did not have a formal diagnosis of autism, as long as they considered their children to be autistic. This latter group may not have received a formal diagnosis for several reasons. First, some families of young children may have been advised to wait until they were older for a formal evaluation, when a diagnosis is more likely to be stable. Second, some families may have received informal suggestions that their child may have been autistic, such as ‘it looks like autism’, but no formal evaluation was pursued. Third, some families may have faced obstacles such as travel constraints or financial actions that limited access to a formal evaluation as most of the health centres are located in the capital city, and most qualified professionals tend to be in the main cities (Romero-Alvarez et al., 2023). Fourth, some families may have recognized their child’s autistic characteristics through professional or parental opinions and not sought or received a formal diagnosis. It is crucial for future research to explore help-seeking behaviours in more depth as families in Ecuador whose children do not have a formal diagnosis of autism are not able to access public services or support in public or private institutions (e.g. schools). As such, barriers in access to diagnosis in Ecuador represent an important target for efforts in research and practice.

### From first concerns to identification and diagnosis

Families initially suspected that their child may be developing atypically based on concerns centred around language and social interaction, which is consistent with broader literature on this topic (Blacher et al., 2019; Crane et al., 2016; De Giacomo & Fombonne, 1998). Families in Ecuador tended to search for professional help soon after they noticed these early signs (e.g. within 6 months), typically when the child was 1–2 years old, which is also consistent with recent studies (Bent et al., 2020) and earlier than in past studies (Crane et al., 2016), suggesting that awareness among parents about the early signs of autism may have increased in recent years. Families tended to address their first concerns to paediatricians, but these concerns were not always followed up. These ‘passive’ responses, which have also been described in other contexts (Bent et al., 2015; Crane et al., 2016), could be attributed to several factors in the Ecuadorian context. These may include professionals’ limited time and resources to facilitate referrals, and/or professionals’ limited self-confidence in screening practices; both of which have previously been described in Ecuadorian paediatric settings (Buffle et al., 2022) but also in other contexts internationally (Crane et al., 2019; Randall et al., 2018; Unigwe et al., 2017).

Families in our study mainly received their children’s diagnosis and follow-up appointments in private consultations, suggesting that autism-specific diagnostic services within the national health service in Ecuador may not be available or visible despite efforts to develop diagnostic units within the national health system in recent years (Buffle, 2021). Notably, nearly half of the respondents, from a variety of socioeconomic backgrounds, opted for assessments in the private health sector, paying out-of-pocket. This finding is significant given that access to services in the public sector requires a diagnostic assessment from a public health service. While our data do not specify the reasons behind the choice to seek a private assessment, this finding could partly explain why parents often consult multiple professionals (i.e. they might initially seek a private assessment followed by confirmation of the assessment within a public health system in order to access support and services). As approximately 6.8% of the Ecuadorian population cover health expenses through private insurance, and 40.5% pay for health services out-of-pocket (World Health Organization, 2016), having diagnoses both in the public and private sectors implies an inefficient use of resources.

It was notable that nearly half of the respondents received a diagnosis of autism from the first professional consulted, but 97% of families sought further consultations. In addition to the aforementioned issues around seeking assessments in both the public and private sectors, another possible explanation for this finding is that many children in the sample had additional needs, not all of which were formally recognized with an autism diagnosis. Further studies should explore the impact of co-occurring difficulties (Lai et al., 2019) and families’ unmet support needs in greater depth.

It is also important to highlight that it was not possible to confirm whether the professionals consulted contributed to clarifying the path towards diagnosis. For instance, families might receive a professional opinion suggesting the possibility of autism yet may not have been given a definitive diagnosis. This uncertainty may drive families to seek opinions from various professionals, leading to a diagnostic odyssey. In other instances, families may have received an autism diagnosis from the first professional consulted. However, if this diagnosis lacked explanation regarding the child’s needs and available educational support, families may have felt compelled to seek out other professionals who can provide such support. Examining families’ pathways in more depth, particularly with a focus on the reasons for their often lengthy and fraught journeys, is an important avenue for future research.

### Post-diagnostic support

As per previous research (Crane et al., 2016, 2018; Montiel-Nava et al., 2020; Paula et al., 2020), families in our sample reported that they received limited post-diagnostic support. While families often received a follow-up appointment from the professional who provided the diagnosis, this appointment tended to be obtained privately. A range of support was provided post-diagnosis, such as language therapy, early intervention and an explanation of their children’s difficulties. However, from the data we have, it is not possible to comment on the strength of the evidence underpinning the interventions provided to families, and/or whether these interventions correspond to children’s needs. This issue may be especially important to address in future research, given that families relied heavily on the Internet and other families as key sources of information. However, much online information about autism may be inaccurate and/or overgeneralised (Aragon-Guevara et al., 2023). Overall, there is clearly a need for evidence-based, freely available information to support families of newly diagnosed autistic children in Ecuador.

### Parental emotions, thoughts and perceptions at diagnosis and in relation to their child being autistic

Many families reported negative emotions when asked about their experience of accessing a diagnosis and of supporting their autistic child. These responses appeared to be underpinned by the lack of information and services for families of autistic children in Ecuador. However, positive emotions were also noted, which were attributed to factors such as the diagnosis affording a better understanding of their children’s needs. These findings align with other studies on the positive impact of an autism diagnosis (Jacobs et al., 2020), despite a general lack of satisfaction with post-diagnostic support (e.g. Crane et al., 2016). An important clinical implication of these findings is the need for professionals to recognize the emotional and practical challenges parents face during the journey towards a diagnosis. Notably, there was a strong sense of families wanting to learn about autism and having a primary role in responding to their child’s needs. These findings align with studies demonstrating the benefits of parent empowerment programmes and parental training (Divan et al., 2015; Green et al., 2010), and it will be important to consider how to adapt existing programmes to the Ecuadorian context.

In addition, fostering a collaborative interaction between parents and professionals is essential, as parents are proactive and eager to learn, wanting to play a primary role in addressing their child’s needs.

### Broader cultural implications

The families who took part in our survey noted their strong reliance on broader family for support. This finding may be particularly relevant in Latin American contexts, where family members provide important long-term support (Campos et al., 2014), and where family connectedness and the obligation of working together to support individual family members’ needs have been described (Halgunseth et al., 2006). It will therefore be important for any developed resources to be applicable beyond primary caregivers, and extent to the broader family unit as well.

Notably, concerns related to stigma, which can impact demand for autism-related support and services, were only reported by a small percentage of participants in this study. This finding contrasts with results from other studies, which have reported high levels of stigma among families of autistic children in Latin America (Montenegro et al., 2022; Paula et al., 2020), and in other countries (Kinnear et al., 2016) along with a reluctance to seek help and support (Divan et al., 2021; Russell & Norwich, 2012; Sakai et al., 2019; Tait et al., 2016). This finding warrants further exploration.

### Strengths and limitations

This study represents the first large-scale investigation into families’ experiences of accessing an autism diagnosis and associated support in Ecuador, highlighting the broad range of journeys and emotions that families face as they come to learn that their child is autistic. The study benefitted from making culturally appropriate adaptations to existing surveys with the support of a parents association in Ecuador, as well as through adopting a diverse, inclusive and comprehensive recruitment strategy (e.g. advertising via radio). Nevertheless, this work is foundational in nature, largely utilizing descriptive analyses. It is crucial for subsequent work to explore aspects of families’ journeys in greater depth, with a focus on using this knowledge to develop appropriate support and services to meet families’ needs.

A key weakness of our study related to the sample. At the time of the survey, Ecuadorian families living in rural areas represented 37% of the national population (National Institute of Statistics and Censuses of Ecuador, 2022), yet in our sample they represented only 6% of participants. This outcome was despite efforts to disseminate information about the study via channels such as radio. One possible explanation for the lack of participation from rural areas was limited access to WiFi to complete the survey. Indeed in 2022, 70.1% households ac cessed this service in urban areas, compared to only 38% of rural families (Instituto nacional de estadísticas y censo, 2022). An alternative explanation relates to the problematic geographic distribution of health centres in Ecuador (Romero-Alvarez et al., 2023), which may have limited help-seeking behaviours among families from rural areas, due to the scarcity of specialist services at a reachable distance. A scarcity of public health centres offering autism services is reflected in our sample, with 67% reporting paying for evaluation services themselves. Further work should consider in-depth qualitative work with families in rural areas, to better understand the barriers and facilitators to support that they face, to inform services and supports.

### Recommendations

At the time of writing, the Ecuadorian guide for clinical practice on autism, initially published in 2017 by the Ministry of Public Health, was being amended. Based on our findings, we suggest that it will be important to use this opportunity to create a more consistent diagnostic process for families. As a single diagnostic pathway may overwhelm health sub-systems and increase inefficiencies, it may be beneficial to explore how to ensure that all actors involved know the multiple pathways to receiving a diagnosis and to providing high-quality services to families (Brian et al., 2019). To define diagnostic pathways in Ecuador, drawing on lessons learned from other countries with similar needs (Brian et al., 2024) could be beneficial. However, the standardization of any procedure across the entire country would require the establishment of a national autism strategy covering key areas related to identification, diagnosis and post-diagnosis services. A national autism strategy should also include healthcare, education and employment support that considers the particular and diversified needs of autistic people across the lifespan. Recommendations within such a strategy could include the following:

1. A Clear Path to Assessment and Diagnosis: Streamlined processes should help families understand the steps they need to take to get an assessment for their child, obtain a diagnosis, and access appropriate services. This path should take into account factors such as the family’s location, knowledge of typical and atypical development, education level and socioeconomic status.
2. Access to Early Identification: This service is crucial for the well-being of both the child and the family, as it provides a rapid understanding of the child’s needs, which could have an impact on the child and broader family’s well-being.
3. Utilize All Available Resources to reduce the burden on the public system, particularly in urban areas.
4. Explore the Use of Solutions for Children Living in Rural Areas, such as mobile developmental clinics that can travel to remote communities, video-conference consultations that can be implemented in a culturally sensitive way and public efforts to build local capacity to allow access to diagnostic and support services.
5. Appropriate Use of Professionals: Allocating specialists for more complex cases while leveraging the skills of family doctors and community doctors, particularly in rural regions, who were notably absent from the identification and diagnosis process, according to families in this survey.
6. Use a Holistic Approach: Addressing healthcare, education and social care needs, considering the diverse and specific requirements of autistic people across the lifespan.
7. Standardize Training and Practices: Training professionals within the public sector to standardize identification and diagnosis practices. This endeavour will ensure consistent, high-quality services across all areas, reducing the need for parents to visit multiple professionals in search of a qualified diagnosis.

It is essential that stakeholder involvement underpins all such endeavours.

## Conclusion

The findings of this study suggest that families of autistic children in Ecuador, much like families of autistic children globally, are quick to notice initial signs of atypical development (e.g. in language and social interaction) and that they seek professional help soon after. However, many families in Ecuador may encounter delays and inconsistencies in obtaining a formal diagnosis due to barriers unique to low- and middle-income countries, such as travel constraints and limited visibility of public services. Addressing this issue is crucial since those who are unable to access a formal diagnosis in Ecuador may have limited access to support. Our findings that families often consult multiple professionals to confirm their children’s diagnosis, and/or consult multiple professionals to seek further explanations and support highlight a need for clear diagnostic pathways in Ecuador. Much like in other countries in the world, post-diagnostic support for families of autistic children appears to be limited in Ecuador, and families rely on the Internet as a source of support. Yet families show a strong desire to learn about autism and how to support their children, suggesting a need for accessible evidence-based information and parent training programmes. Notably, reliance on extended family for support is significant in the Ecuadorian context, emphasizing the importance of resources that engage the broader family unit. From our findings, we make a number of recommendations that may form part of an autism strategy in Ecuador (e.g. clarifying paths to autism assessment and diagnosis). The involvement of stakeholders will be crucial in further developing and refining such recommendations.

## Supplemental Material

sj-docx-1-aut-10.1177_13623613241281029 – Supplemental material for Journeys towards accessing an autism diagnosis and associated support: A survey of families of autistic children in Ecuador Supplemental material, sj-docx-1-aut-10.1177_13623613241281029 for Journeys towards accessing an autism diagnosis and associated support: A survey of families of autistic children in Ecuador by Paulina Buffle, Thalia Cavadini, María de Lourdes Ortega, Cristina Armijos, Patricia Soto, Edouard Gentaz and Laura Crane in Autism
