# Supplementary material for: Journeys towards accessing an autism diagnosis and associated support: A survey of families of autistic children in Ecuador
Source: Autism. 2024 Sep 28;29(3):596–613. doi: 10.1177/13623613241281029 (PMC11894907; doi:10.1177/13623613241281029)
Supplement: sj-docx-1-aut-10.1177_13623613241281029 – Supplemental material for Journeys towards accessing an autism diagnosis and associated support: A survey of families of autistic children in Ecuador [file sj-docx-1-aut-10.1177_13623613241281029.docx]

**Supplementary materials**

**Supplementary Materials 1:** Participants’ further characteristics

|  |  | Total sample | With a formal autism diagnosis | Without a formal autism diagnosis |
| --- | --- | --- | --- | --- |
|  |  | (*n*=767) | (*n*=651) | (*n*=116) |
|  |  | n (%) | n (%) | n (%) |
| Other children or people with special needs under their care | | | | |
|  | Yes | 117 (15%) | 96 (15%) | 21 (18%) |
|  | No | 650 (85%) | 555 (85%) | 95 (82%) |
| Participant has a diagnosis of autism or Asperger syndrome | | | | |
|  | Yes | 78 (10%) | 65 (10%) | 13 (11%) |
|  | No | 686 (89%) | 586 (90%) | 100 (86%) |
|  | No answer (NA) | 3 (<1%) | 0 | 3 (3%) |
| Participant ‘thinks’ he/she might be autistic | | | | |
|  | Yes | 170 (22%) | 146 (22%) | 24 (21%) |
|  | No | 594 (77%) | 504 (77%) | 90 (78%) |
|  | NA | 3 (<1%) | 1 (<1%) | 2 (2%) |
| Member of a parent’s support group | | | | |
|  | Yes | 501 (65%) | 429 (66%) | 72 (62%) |
|  | No | 264 (34%) | 221 (34%) | 43 (37%) |
|  | NA | 2 (<1%) | 1 (<1%) | 1 (1%) |
| Sole caregiver (e.g. single parent) | | | | |
|  | Yes | 200 (26%) | 172 (26%) | 28 (24%) |
|  | No | 565 (74%) | 479 (74%) | 86 (74%) |
|  | NA | 2 (<1%) | 0 | 2 (2%) |

**Supplementary Materials 2:** Additional information on community involvement

The panel included one representative and one member of the Ecuadorian Parent’s Association of the Autism Spectrum APADA. It also included two parents of autistic children who were not members of a parents’ association. one special educator, one paediatrician with experience in developmental paediatrics, and one psychologist with experience in autism early intervention. The panel provided feedback and suggestions on the following points: the need to collect information about children’s current developmental difficulties, parents’ autism diagnostic status, and single parental status.

They also provided feedback on the variability of actions taken by parents after the first concerns, the variability of professionals receiving parental first concerns, the variability of professionals providing a diagnosis, possible sources of financing the diagnosis, variability of diagnosis labels, variability types of post-diagnosis support, variability interventions, treatment and therapies, the terminology used in the questions and items related to employment situation and income level. They also provided feedback on open-ended phrasing questions designed to obtain in-deep information on parents’ experiences. Parents suggested avoiding questions containing words related to “helpless”, “worry”, “problem”, “discrimination”, and “stigma,” to avoid potentially inducing negative feelings in caregivers. Finally, the panel provided advice on the inclusion of a question related to the child’s current strengths and interests, aiming to end the survey on a positive note.

**Supplementary Materials 3:**  The questionnaire

**Section 1: About you**

1 Your relationship with the person with autism

- I am his/her mother
- I am his/her brother
- I am his/her grandfather/grandmother
- I am his/her aunt/uncle
- I am his/her father
- I am his/her guardian
- I am his/her stepmother/stepfather

2 Do you have other children or people with special needs under your care?

- Yes
- No

3 Have you been diagnosed with autism or Asperger syndrome?

- Yes
- No

4 Do you think you are on the autism spectrum?

- Yes
- No

5 Are you currently a member of a support group for parents of individuals with autism?

- Yes
- No

**Section 2: About your demographics**

6 Your age

- 18-29 years
- 30-39 years
- 40-49 years
- 50-59 years
- 60 or older

7 Are you a single parent or caregiver to your autistic child?

- Yes
- No

8 Your ethnic origin

- Mestizo
- White
- Indigenous
- Afro-descendant

9 How would you describe your current employment situation?

- I work (I have a salary)
- Work (I am a freelance)
- Work (I work a few hours a week)
- I don't work (but I'm looking for work)
- I do not work (another member of the family works to cover the needs)
- I do not work (I have a disability)
- I don't work (I stopped working to take care of my son with autism)
- Prefer not to answer

10 Your highest level of education

- Primary
- High school
- Technical degree
- Craft certificate
- Academic
- Master's or doctorate

11 How much do you estimate your income level per month, based on what you earned in the last 2 years?

- Prefer not to answer
- Less than $425
- $427 to $750
- $750 to $2000
- $2,000 to $2,500
- More than $5000

12 How many people, including you, live in the household with that income?

- 1
- 2
- 3
- 4
- 5 or more

13 How do you define your economic situation?

- Very low
- Low
- Middle-low
- Middle income
- Middle-High
- high
- Very high

**Section 3: About your child**

14 Current age of your child with autism (if you have more than 1 child with autism, answer about the youngest)

- Less than 2 years
- Between 2 and 3 years
- Between 4 and 6 years
- Between 7 and 9 years
- Between 10 and 12 years
- Between 13 and 15 years old
- Between 16 and 18 years
- Between 18 and 29 years
- 30 or more

15 Your child with autism lives in:

- The city - close to a city
- In the countryside - far from a city

16 The gender of your child with autism

- Male
- Female
- Other

17 If your child with autism has siblings, how many of them live with him/her?

- 0
- 1
- 2
- 3 or more

**Section 4: About your first concerns and consultations**

18 You **first had developmental concerns** when your child was:

- Less than 5 months
- Between 6 and 12 months
- Between 1 and 2 years
- Between 3 and 4 years
- Between 5 and 6 years
- Between 7 and 8 years
- Between 9 and 10 years
- Between 11 and 12 years
- Between 13 and 18 years old
- Over 18 years, tell us how many: ________________________________________________

19 Why **did** you start to worry about your child? (You can choose several)

- Delay in starting to speak
- Delay in skills (for example, in walking)
- Difficulty developing the game as children their age do
- Difficulty in social development (i.e., difficulty relating to other people as children their age do)
- He paid no attention when he was called by the name
- Behavior problems (eg, hyperactivity or tantrums)
- Trouble learning at school
- did not communicate with us
- Rituals / obsessions / hated changes changes / too much attachment to certain objects
- Did not obey simple instructions
- Mental health difficulties
- Medical problems
- Sleeping difficulties
- Eating difficulties
- Sensitivities to sound, light, water, other thing(s). Explain which ones:
- Other (explain) ________________________________________________
- We had no concern until a professional raised our concerns.

20 At that time, **did** you **take your son** to see a professional?

- Yes, I took him/her to consult a professional from the public network
- Yes, I took him/her to consult a private professional
- No, because I didn't know where to take him/her
- No, for another reason (Explain the reason) _______________________________________

21 How much time passed between your first concerns and the **FIRST time you took your child** to see a professional?

- I took him/herimmediately
- I took him/her after 1 and 2 months
- I took him/her after 3 and 6 months
- I took him/her after 7 and 12 months
- more than 1 year after first concerns

22 Does your child **have a formal diagnosis of some form of autism** from a professional in the public or private health network?

- Yes
- No, but we suspect that he has some form of autism

*Skip to: End of Survey if “Does your child* ***have a formal diagnosis of some form of autism*** *from a professional in the public or private health network? = No*

23 Was your child's formal diagnosis of autism given in Ecuador? = No

- Yes
- No

*Skip to: End of Survey if “Was your child's formal diagnosis of autism given in Ecuador?*

24 Does your child with a formal diagnosis of autism currently reside in Ecuador?

- Yes
- No

*Skip to: End of Survey if “Does your child with a formal diagnosis of autism currently reside in Ecuador?”*

25 Who was the FIRST professional to see your child?

- Pediatrician
- Child Psychiatrist / Psychiatrist
- Psychologist
- Neurologist / Neuropediatrician
- Dispensary doctor
- School professional
- Social worker
- I'm not sure or I don't know
- Other, specify: ________________________________________________

26 What happened during the consultations with the FIRST professional?

- The professional provided any diagnosis
- The professional said "no problem" or "don't worry" or "it will pass with age"
- Other (specify) ________________________________________________

27 If your child received a diagnosis in the consultations with the FIRST professional, **what was the diagnosis**?

- Autism
- Asperger syndrome
- Autism spectrum disorder
- He/she had “eary signs” of autism (in children under 3 years old)
- Language disorder
- Developmental delay
- Generalized development disorder
- That the child had some difficulty without saying what
- Other diagnosis, specify which: ________________________________________________
- There was no diagnosis with the FIRST professional

28 To get a formal diagnosis, did you need to see a SECOND professional?

- Yes
- No

29 Who was the SECOND professional?

- Pediatrician
- Child Psychiatrist / Psychiatrist
- Psychologist
- Neurologist / Neuropediatrician
- Dispensary doctor
- School professional
- Social worker
- I'm not sure or I don't know
- Other, specify: ________________________________________________

30 What happened during the consultations with the SECOND professional?

- The professional provided any diagnosis
- The professional said "no problem" or "don't worry" or "it will pass with age"
- Other (specify) ________________________________________________

31 If your child received a diagnosis in consultations with the SECOND professional, what was the diagnosis?

- Autism
- Asperger syndrome
- Autism spectrum disorder
- Who has warning signs of autism (in children under 3 years old)
- Language disorder
- Developmental delay
- Generalized development issue
- That the child had some difficulty without saying what
- Other, specify which) ________________________________________________
- There was no diagnosis with the SECOND professional

32 To have a formal diagnosis of autism, how **many professionals has your child seen since you had your first concerns** ?

- 1
- 2
- 3
- 4 or more

33 How **old was your child at the time of the formal diagnosis of autism** ?

- Less than 36 months (we were told my child had warning signs of autism)
- Between 3 and 4 years
- Between 5 and 6 years
- Between 7 and 8 years
- Between 9 and 10 years
- Between 11 and 12 years
- Between 13 and 18 years old
- Over 18 years, tell us how old: ________________________________________________

34 The diagnosis was given:

- In a written report
- They told us orally (not written)

35 Who was the person who gave the final diagnosis of autism?

- Paediatrician
- Child Psychiatrist / Psychiatrist
- Psychologist
- Neurologist / Neuropediatrician
- Dispensary doctor
- I'm not sure or I don't know
- Other, specify: ________________________________________________

36 At that time, were you expecting the diagnosis received?

- Yes
- No

37 In which **province or country** was your child formally diagnosed with autism?

________________________________________________________________

38 In which **province or country** was your child LIVING in at the time?

________________________________________________________________

39 The diagnosis was:

- Paid for by parents
- Covered by private insurance
- Given in the public insurance system (IESSS)
- Given in institutional insurance systems (ISSFA, ISSPOL)
- Made in the public health network ( MSP)
- Covered by a charity (Foundation)
- Other. Explain: ________________________________________________

40 Has your child ever been formally diagnosed **with any other condition**?

- Yes, a physical disability (including epilepsy, hearing impairment, visual impairment)
- Yes, a learning disorder (including dyslexia and general learning disabilities)
- Yes, an affective condition (including depression and anxiety)
- Yes, a behavioural condition (including ADHD, Tourette syndrome)
- Yes, a mental health condition (including bipolar disorder, OCD, schizophrenia)
- Yes, a genetic condition (including fragile X syndrome)
- Yes, another condition. Tell us which: ____________________________________________
- None of the above

41 Does your child have any other undiagnosed difficulty?

- Digestive difficulties
- Sleep difficulties
- Sensory difficulties
- Behavior difficulties
- Eating disorders
- Intellectual difficulty
- Does not speak
- Other (Explain which): ________________________________________________

42 Could you choose a few words to tell us what your emotions and thoughts were when you received the diagnosis?

___________________________________________________

43 At the time of the diagnosis, were you provided with suggestions about professionals or associations that can monitor/support your child?

- They provided me with the information of a professional so that I can organize the appointments for the follow-up of my son
- I am not sure if I have received suggestions for the professional follow-up of my child
- I did not receive information

44 If, after diagnosis, your child had follow-up appointments for autism, what did they consist of? (you can choose several answers)

- Explanations about your child's difficulties
- Early intervention for autism
- Behavioral intervention for autism
- Language therapy
- Parent Training
- Sensory therapy
- Motor therapy
- Other. Please indicate which ________________________________________________
- Had no follow-up

45 If your child had follow-up appointments after their diagnosis, these were:

- In the Ecuadorian public sector
- In the Ecuadorian private sector
- In both

46 Who recommended the form of follow-up for your child received after diagnosis? (you can choose several answers)

- The professional who made the diagnosis
- Other parents
- Teachers
- Internet
- Friends
- Yourself
- Other (Explain who:) ________________________________________________
- Does not apply, he/she did not have a follow-up

47 After the diagnosis of autism, did your child also receive MEDICAL treatment for any illness?

- No
- Yes. Tell us which: ________________________________________________

48 To receive any care for your child, did you have to move to another province or city?

- Yes
- N

49 What OTHER supports, apart from the support offered by professionals**,** have you received after your child's diagnosis? (You can choose several)

- General tips for raising my child with autism
- Contact with other parents of autistic children
- Contact with a charity
- Help from a preschool/school institution to be admitted
- Help to obtain a disability card
- Help with his/her medical problems
- State economic direct transfer (bonds)
- Other. Please indicate which ________________________________________________
- None

50 **Who has been the most important support** for you and your child?

- Grandparents
- Aunts, uncles and extended family
- Friends
- Group in social networks
- Religious group
- Charities / ONGs
- Public sector professionals (indicate the type profession) ______________________
- Professionals from the private sector (indicate the type profession) ________
- Other ___________________

51 Please choose a few words to explain what it means to you that your child has autism

________________________________________________________________

52 What are your child's most common interests right now?

________________________________________________________________

53 What are your child's straights and talents right now?

________________________________________________________________

54 If there was something particularly important to you in relation to therapies or treatments, education and please choose a few words to explain:

________________________________________________________________

**Supplementary Material 4:** Age, onset, nature, and caregiver's response to initial developmental concerns.

|  |  | All respondents (*n*=767) | | Sample with a formal diagnosis (*n*=651) | | Sample without a formal diagnosis (*n*=116) | | |
| --- | --- | --- | --- | --- | --- | --- | --- | --- |
|  |  | *n* (%) | *n* (%) | | | | *n* (%) |  |
| Age at initial developmental concerns | |  |  | | | |  |  |
|  | Less than 5 months old | 67 (9%) | 63 (10%) | | | | 4 (3%) |  |
|  | Between 6 and 12 months old | 135 (18%) | 114 (18%) | | | | 21 (18%) |  |
|  | Between 1 and 2 years old | 361(47%) | 302 (46 | | | | 59 (51%) |  |
|  | Between 3 and 4 years old | 134 (17%) | 119 (18%) | | | | 15 (13%) |  |
|  | Between 5 and 6 years old | 26 (3%) | 17 (3%) | | | | 9 (8%) |  |
|  | Between 7 and 8 years old | 16 (2%) | 14 (2%) | | | | 2 (2%) |  |
|  | Between 9 and 10 years old | 11 (1%) | 8 (1%) | | | | 3 (3%) |  |
|  | Between 11 and 12 years old | 15 (2%) | 12 (2%) | | | | 3 (3%) |  |
|  | Between 13 and 18 years old | 0 | 0 | | | | 0 |  |
|  | Over 18 years old | 0 | 0 | | | | 0 |  |
|  | NA | 2 (<1%) | 2 (<1%) | | | | 0 |  |
| Nature of initial concerns | |  |  | | | |  |  |
|  | Language delay | 507 (66%) | 435 (67%) | | | | 72 (62%) |  |
|  | Did not respond to name | 426 (56%) | 369 (57 %) | | | | 57 (49%) |  |
|  | Difficulties in social interaction | 421 (55%) | 352 (54%) | | | | 69 (59%) |  |
|  | Did not played as expected | 366 (48%) | 318 (49%) | | | | 48 (41%) |  |
|  | Did not follow or understand simple instructions | 353 (46%) | 301 (46%) | | | | 52 (45%) |  |
|  | Behaviour difficulties (e.g. hyperactivity, tantrums) | 347 (45%) | 291 (45%) | | | | 56 (48%) |  |
|  | Sensory sensitivity | 312 (41%) | 270 (41%) | | | | 42 (36%) |  |
|  | Did not communicate with adults | 283 (37%) | 248 (38%) | | | | 35 (30%) |  |
|  | Rituals/obsessions/intolerance to change/particular object attachments | 254 (33%) | 221 (34%) | | | | 33 (28%) |  |
|  | Sleep problems | 233 (30%) | 203 (31%) | | | | 30 (26%) |  |
|  | Delay in other milestones (e.g. walking) | 198 (26%) | 162 (25%) | | | | 36 (31%) |  |
|  | Eating problems | 191 (25%) | 165 (25%) | | | | 26 (22%) |  |
|  | Learning difficulties at school/preeschool | 129 (17%) | 102 (16%) | | | | 27 (23%) |  |
|  | Medical problems | 96 (13%) | 83 (13%) | | | | 13 (11%) |  |
|  | Mental health difficulties | 41 (5%) | 26 (4%) | | | | 15 (13%) |  |
|  | Parents had no concerns until a professional raised concerns | 72 (9%) | 58 (9%) | | | | 16 (14%) |  |
|  | Motor atypicities (atypical postures, tip-toe walking, crab walking), (flapping, rocking or repetitive movements), late development or hypotonia | 18 (2%) | 16 (2%) | | | | 3 (3%) |  |
|  | Seizures or language regression | 12 (2%) | 11 (2%) | | | | 1 (1%) |  |
|  | Emotion development (e.g. excessive irritability, extreme fears, no smiling, laughing alone) | 7 (1%) | 5 (1%) | | | | 2 (2%) |  |
|  | Othe not specidied | 13 (2%) | 13 (2%) | | | | 2 (2%) |  |
|  | NA | 2 (0%) | 1 (1%) | | | | 1 (1%) |  |
| Caregiver's reactions to initial concerns | |  | | |  |  |  |  |
|  | Consulted a private professional | 467 (61%) | 407 (63%) | | | | 60 (52%) | |
|  | Consulted a professional from the public network | 236 (31%) | 199 (31%) | | | | 37 (32%) | |
|  | Did mot consult for another reason | 29 (4%) | 20 (3%) | | | | 9 (8%) | |
|  | Did not know where to consult | 24 (3%) | 15 (2%) | | | | 9 (8%) | |
|  | NA | 11 (1%) | 10 (2%) | | | | 1 (1%) | |
| Time from initial concern to first professional consultation | |  |  | | | |  | |
|  | Immediately | 240 (31%) | 205 (31%) | | | | 35 (30%) | |
|  | Between 1 and 2 months had passed | 166 (22%) | 145 (22%) | | | | 21 (18%) | |
|  | Between 3 and 6 months had passed | 161 (21%) | 140 (22%) | | | | 21 (18%) | |
|  | More than 1 year had passed | 119 (16%) | 99 (15%) | | | | 20 (17%) | |
|  | Between 7 months and 1 year had passed | 75 (9%) | 57 (9%) | | | | 18 (16%) | |
|  | NA | 11 (1%) | 10 (2%) | | | | 1 (1%) | |

**Supplementary Materials 5:** Child age at the moment of diagnosis (n=651)

|  | | *n (%)* |
| --- | --- | --- |
|  | Less than 36 months (“Early signs” of autism early detected) | 205 (31%) |
|  | Between 3 and 4 ears old | 249 (38%) |
|  | Between 5 and 6 ears old | 81 (12%) |
|  | Between 7 and 8 ears old | 40 (6%) |
|  | Between 9 and 10 ears old | 19 (3%) |
|  | Between 11 and 12 ears old | 20 (3%) |
|  | Between 13 and 18 ears old | 28 (4%) |
|  | Over 18 years old (between 20 and 30 years old) | 9 (1%) |

**Supplementary Materials 6:** Geographic location at the time of diagnosis (n=642)

| Province | n (%) |
| --- | --- |
| Pichincha | 399 (62%) |
| Guayas | 127 (20%) |
| Manabí | 28 (4%) |
| Azuay | 27 (4%) |
| Loja | 15 (2%) |
| Chimborazo | 12 (2%) |
| Esmeraldas | 11 (2%) |
| Tungurahua | 8 (1%) |
| Ecuador | 4 (1%) |
| Santo Doming Tsachilas | 3 (<1%) |
| Imbabura | 2 (<1%) |
| Morona Santiago | 2 (<1%) |
| Carchi | 1 (<1%) |
| El Oro | 1 (<1%) |
| Los Rios | 1 (<1%) |
| Sucumbios | 1 (<1%) |

**Supplementary Materials 7:** Parental emotions and thoughts and thoughts when receiving a formal diagnosis for their children, further examples (n=630)

| **Categories** | Examples |
| --- | --- |
| **Overwhelming negativity** (n=403; 64%). | “Preoccupation and anguish.”  “I felt desolation and frustration.”  “I felt depressed." |
| Helplessness and a lack of understanding about autism (n=69; 11%). | “I felt helpless, but I immediately asked how I could help him.”  “I felt ignorance, frustration and impotence for not knowing how to help him, not knowing where to start.” |
| ‘Bittersweet’: a stressful journey but a clear outcome (n=69; 11%). | “It was a bittersweet feeling because what was happening to my son already had a name, (…), and at the same time we had the tools to help him and a path of light and hope was opened.”  “I felt total bewilderment, but it was important to finally know what was happening with my daughter's health.” |
| No more uncertainty: relief and awareness (n=57; 9%). | “I felt calm knowing at last what was wrong with him”  “Peace of mind, the security of knowing where to start with the right accompaniment for my beloved child.” |
| What next? Wanting to help their child (n=32; 5%). | "At that moment I thought about looking for information about autism and looking for a way to help my daughter”  “Well, I thought, 'now I know what my daughter has, I must seek to learn so that I can help and understand her.” |

**Supplementary Material 8.** Perceptions and understanding of their child’s autism diagnosis, further examples (n=621; 1031 units of responses)

| Categories | Examples |
| --- | --- |
| Category 1: Mixed emotions (n=526; 51%) | |
| Need for patience and tolerance (n=185; 18%) | “It represents for me that I must take care of her and treat her with much more patience”  “Acceptance of differences” |
| Gaining strength, courage and perseverance to fight for their child (n=341; 33%) | “It means a constant fight, never give up, be more empathetic |
| Category 2: Negative emotions (n=296; 29%) | |
| 2.1 Sadness, pain, and desperation; for the present and future (n=104; 10%) | “The hardest thing in my life”  “It represents pain, frustration, anguish for not knowing what to do” |
| 2.2 Fear and uncertainty (n=90; 9%) | “A constant concern since he is an adult and has serious behaviour problems”  “Anxiety about thinking what will happen when I am no longer here. A great uncertainty for his safety and future.” |
| 2.3 Concerns about accessing support (n=73; 7%) | “It is difficult, since there are not many schools that accept him or can support him, as well as the cost of therapies”  “A full-time hard work” |
| 2.4 Exclusion and discrimination (n=29; 3%) | “(…) It represents having to make of my daughter the most functional and independent person to face of a complex and selfish world”  “Society does not understand anything, it only excludes" |
| Category 3: Positive emotions (n=205; 20%) | |
| 3.1 A path forward (n=93; 9%): | “It represents the engine to move forward for him”  “A great responsibility to seek information and support to learn about ASD and thus be able to help my son”  “It means learning about autism to know how to guide him in the best way of respecting and loving him.”  “My son's autism has made me a more patient, more human person, I continue to educate myself, study, inform myself of scientific advances.” |
| 3.2 My child as a blessing (n=112; 11%) | “Now I see it as a great blessing.”  “It represents union and love” |

**Supplementary Materials 9:** The most important source of support for the child and parents (n=630 participants, 1064 responses recorded)

|  | *n (%)* |
| --- | --- |
| Grandparents and extended familly | 576 (54%) |
| Professionals from the private sector… | 147 (14%) |
| Groups on social media | 107 (14%) |
| Charities | 67 (6%) |
| Friends | 60 (6%) |
| Public sector professionals | 60 (6%) |
| Religious group | 20 (2%) |
| Parent association | 8 (<1%) |
| School | 5 (<1%) |
| Other professionals | 3 (<1%) |
| Other | 11 (1%) |
